# Amoebicidal activity of cationic carbosilane dendrons derived with 4-phenylbutyric acid against *Acanthamoeba griffini* and *Acanthamoeba polyphaga* trophozoites and cysts

**DOI:** 10.1038/s41598-022-19200-w

**Published:** 2022-09-02

**Authors:** P. López-Barona, C. Verdú-Expósito, T. Martín-Pérez, N. Gómez-Casanova, T. Lozano-Cruz, P. Ortega, R. Gómez, J. Pérez-Serrano, I. Heredero-Bermejo

**Affiliations:** 1 Department of Biomedicine and Biotechnology, Faculty of Pharmacy, University of Alcalá, 28871 Alcalá de Henares, Spain; 2 Department of Organic and Inorganic Chemistry, Andrés M. del Río Chemistry Research Institute (IQAR), Ramón y Cajal Health Research Institute (IRYCIS), Bioengineering, Biomaterials and Nanomedicine Networking Research Center (CIBER-BBN), University of Alcalá, 28871 Madrid, Spain

**Keywords:** Antiparasitic agents, Pathogens

## Abstract

Amoebae from the genus *Acanthamoeba* are important pathogens responsible for severe illnesses in humans such as *Acanthamoeba* keratitis and granulomatous amoebic encephalitis. In the last few decades, AK diagnoses have steadily increased. Most patients suffering from AK were contact lens users and the infection was related to poor hygiene. However, therapy is not yet well established, and treatments may last for several months due to resistance. Moreover, these treatments have been described to generate cytotoxicity. Therefore, there is an urgent need to develop new therapeutic strategies against AK. In this study, the amoebicidal activity of different generation cationic carbosilane dendrons derived with 4-phenylbutyric acid was demonstrated against *Acanthamoeba polyphaga* and *Acanthamoeba griffini* trophozoites and cysts*.* In addition, the combination of chlorhexidine digluconate and the most effective dendron (ArCO_2_G_2_(SNMe_3_I)_4_) showed an in vitro effect against *Acanthamoeba* trophozoites and cysts, reducing the minimal trophozoite amoebicidal concentration as well as concentrations with cysticidal activity.

## Introduction

Amoebae from the genus *Acanthamoeba* are ubiquitous protozoa which can be opportunistic pathogens in humans. They are the etiologic agent of *Acanthamoeba* keratitis (AK), a sight-threatening disease related to the use of contact lenses, and granulomatous amoebic encephalitis, a fatal central nervous system disease more common in immunocompromised individuals^1–3^.

The ameba life cycle includes the trophozoite form, which is able to feed, reproduce, and infect, and the cyst stage, which is the resistant form^1–3^. The change from trophozoite to cyst is usually induced by adverse environmental conditions. Cysts can resist extreme environmental pressures such as high temperatures, ultraviolet radiation, gamma radiation and changes in pH, among others^4,5^. Moreover, some drugs used to treat AK induce cyst formation^6^. Therefore, eradication is challenging and requires the development of new treatments.

For AK infections, standard treatment drugs are biguanides (such as chlorhexidine (CLX)), diamidines (such as propamidine isethionate) and the aminoglycoside antibiotic, neomycin^3,7^. Biguanides and diamidines are membrane-acting agents while neomycin is a protein synthesis inhibitor^8^. They are generally used in combination to overcome drug resistance, although their high cytotoxicity along with their inability to eliminate cysts (recurrences occur in about 10% of cases) has led to the search for new compounds with therapeutic applications^7,8^. Although the posology may vary, hourly administration for three days is routinely recommended. Total therapy duration is 3–4 weeks, although patient withdrawal is an additional cause of treatment failure^4^. Furthermore, resistance to standard treatments has been reported, mainly when CLX is used as monotherapy^9^.

In recent years, newly synthesized dendritic compounds have been described as an alternative for treating bacterial, viral, and protozoal infections^10–13^. These compounds have shown anti-*Acanthamoeba* activity against trophozoites and cysts, as well as low cytotoxicity in vitro^11^. Dendritic compounds can be combined with standard drugs used in therapy. Consequently, there would be a reduction in the required effective drug concentrations and in the cytotoxic effects on patients. In some cases, synergy has also been described^14^. Moreover, the strategy of combined therapy with dendritic compounds could contribute to prevent drug resistance development^15^.

In an attempt to identify new therapeutic approaches, our study focused on the evaluation of different generations (1–3) of carbosilane dendrons derived with 4-phenylbutyric acid (PBA) against trophozoites and cysts of *Acanthamoeba spp.* clinical isolates. Additionally, possible synergistic effects of treatments combined with CLX were tested. Cytotoxicity assays were then performed with effective concentrations. Finally, scanning electron microscopy was used to elucidate the effects of the most effective dendritic compound alone and in combination with CLX on trophozoite structure.

## Materials and methods

### *Acanthamoeba spp.* clinical strains

*A. polyphaga* 2961 (kindly supplied by Dr. E. Hadas, Poznan University of Medical Sciences, Poland) and *A. griffini* MYP2004 (isolated by our research group, University of Alcalá, Spain) were used^16^. *A. polyphaga* 2961 was grown at 32 °C in Peptone Yeast Glucose broth supplemented with Bacto Casitone (PYG + B) and *A. griffini* MYP2004 was grown at 37 °C in CERVA^16,17^. The strains were maintained by weekly media changes.

### Dendritic compounds and standard treatment drugs used

**Figure. 1:**
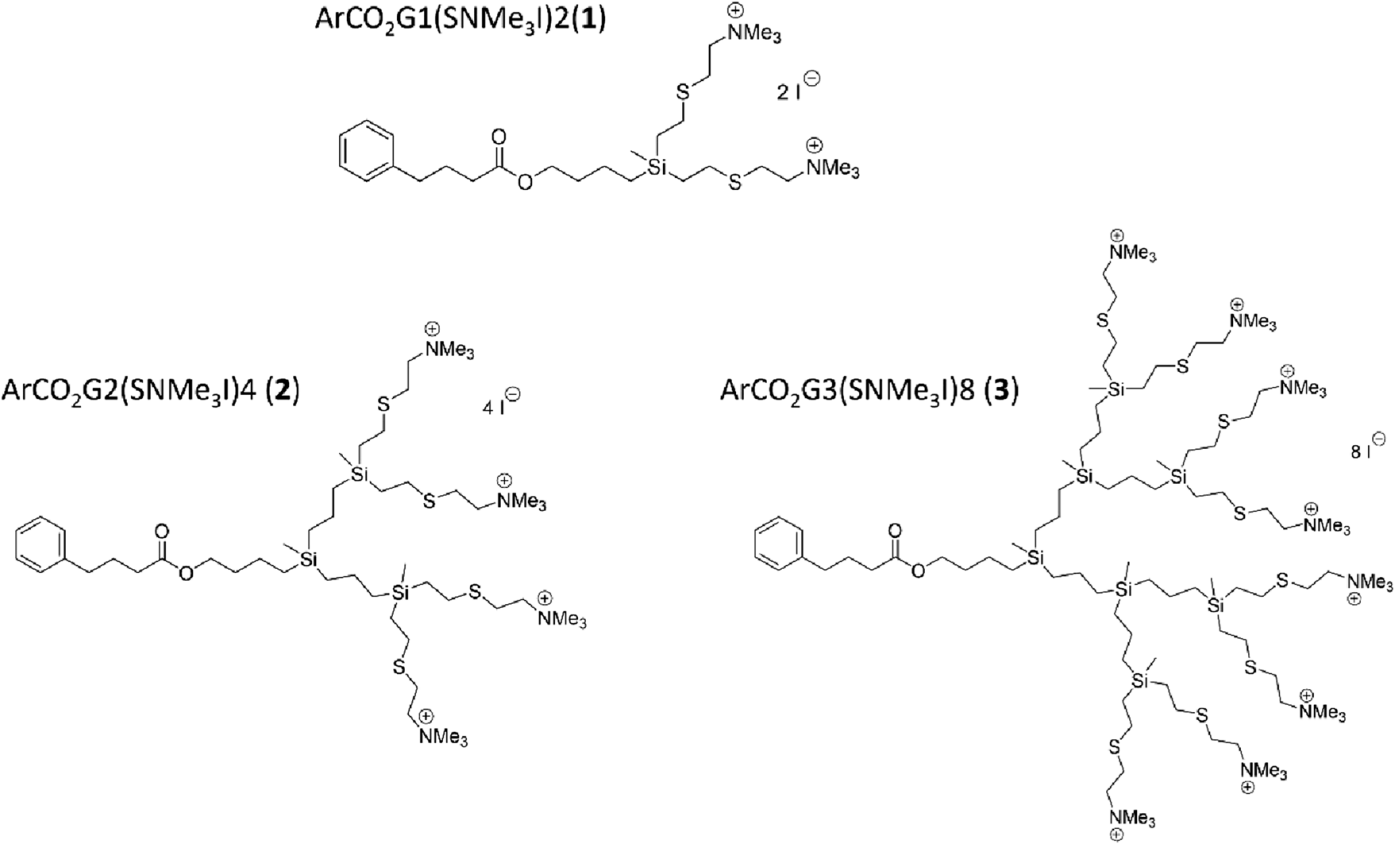
Structures of cationic carbosilane dendrons derived from 4-phenylbutyric ArCO_2_Gn(SNMe_3_I)m, n: generation, m: number of functional groups. (n = 1, m = 2 (**1**): ArCO_2_G_1_(SNMe_3_I)_2_; n = 2, m = 4 (**2**): ArCO_2_G_2_(SNMe_3_I)_4_ and n = 3, m = 8 (**3**): ArCO_2_G_3_(SNMe_3_I)_8_).

Different generations of cationic carbosilane dendrons derived using 4-phenylbutyric acid (PBA) at the ArCO_2_Gn(SNMe_3_I)m focal point (n = 1, m = 2; n = 2, m = 4 and n = 3, m = 8) were studied and are referred to as ArCO_2_G_1_(SNMe_3_I)_2_ (**1**), ArCO_2_G_2_(SNMe_3_I)_4_ (**2**) and ArCO_2_G_3_(SNMe_3_I)_8_ (**3**), respectively (Fig. 1). The focal point is the location from which the reactive peripheral groups emerge to form the hyperbranched wedge. These systems were prepared as described by Lozano-Cruz et al*.,* 2020. To perform combination assays, chlorhexidine digluconate (CLX) (Sigma-Aldrich Ltd., St. Louis, MO, USA) was considered the reference drug^3^.

### Amoebicidal activity assays against trophozoites

The three dendrons, ArCO_2_G_1_(SNMe_3_I)_2_ (**1**), ArCO_2_G_2_(SNMe_3_I)_4_ (**2**) and ArCO_2_G_3_(SNMe_3_I)_8_ (**3**) were evaluated against *A. polyphaga* 2961 and *A. griffini* MYP2004 trophozoites. Assays were prepared in 96-well microtiter plates treated with Poly-L-Lysine (CellStar, Greiner Bio-one).

Firstly, the inoculum was adjusted to 10,000 trophozoites (*A. polyphaga* 2961) and 15,000 trophozoites (*A. griffini* MYP2004) per well, based on a previous strain growth analysis performed in our laboratory. For this purpose, a Fuchs-Rosenthal^©^ counting chamber (Optic Labor) and 0.2% Congo Red in distilled water (Congo Red, Sigma Aldrich) were used under an optic microscope (Carl Zeiss)^17^.

Secondly, the dendrons were twofold serially diluted from stock solutions to reach final concentrations ranging from 2 to 512 mg/L and 100 μL of each concentration were added to each well. Then, 100 μL of the adjusted trophozoite solution (2 × PYG + B and 2 × CERVA for *A. polyphaga* 2961 and *A. griffini* MYP2004, respectively) were also added. Plates were incubated at 32 °C for *A. polyphaga* 2961 and 37 °C for *A. griffini* MYP2004. Manual counting using the 0.2% Congo Red exclusion assay was performed at 24 and 48 h of treatment and percent viability was defined as: % viability = (mean treated/mean control) × 100. The minimum trophozoite amoebicidal concentration (MTAC) was defined as the lowest concentration of test solution that produced a complete reduction in trophozoite viability^18^.

Each drug concentration was tested in triplicate and in at least two independent experiments. A control well containing the amebae with no treatment and control wells of medium were included. All microtiter plates were sealed with Parafilm^®^. These procedures apply to all tests carried out in this study.

### Cysticidal activity assays

Cysts were obtained from ameba cultures in logarithmic phase under optimal growing conditions. The medium was replaced with Neff’s encystment medium and flasks were agitated for 9 days at room temperature^17^. *A. polyphaga* 2961 and *A. griffini* MYP2004 cyst assays were run in 96-well microtiter plates (Deltalab).

The inoculum was adjusted in Neff’s encystment medium, and 10,000 cysts were inoculated per well for both strains. The dendrons were twofold serially diluted to final concentrations ranging from 2 to 512 mg/L; 100 μL of each concentration were mixed with 100 μL of adjusted cyst solution. The dendron solutions were removed after 24 and 48 h of treatment, then each well was washed with 1 × PBS (10x, Sigma Aldrich, St. Louis, MO, USA) and culture medium was added (1 × PYG + B for *A. polyphaga* 2961 and 1 × CERVA *A. griffini* MYP2004). Plates were incubated at optimal growing conditions (32 °C for *A. polyphaga* 2961 and 37 °C for *A. griffini* MYP2004). Wells were observed three times per week with an inverted microscope (Motic AE21) for 21 days to visualize excystment and determine the minimum cysticidal concentration (MCC), defined as the lowest concentration that completely inhibits excystment and trophozoite growth^18^.

### Combined treatment against trophozoites

To perform these assays, the procedure was the same as for the amoebicidal activity assays described above. For this purpose, the checkerboard method was used^19^. The most effective dendron was tested in combination with CLX in a final volume of 200 μL (1:1 ratio). Required concentrations were achieved by serial dilution. Dendron concentrations ranged from 0.5 to 16 mg/L (< MTAC), while CLX concentrations ranged from 0.5 to 4 mg/L (< MTAC). Manual counting was performed after 24 and 48 h of incubation under optimal growing conditions to determine trophozoite viability compared to the untreated control.

To determine synergy, the fractional inhibitory concentration index (FICI) was calculated:

$${\text{FICI }}\left( {{\text{combination}}} \right) \, = \, [{\text{MTAC}}_{{({\text{Dendron}} + {\text{ CLX}})}} /{\text{ MTAC}}_{{\left( {{\text{Dendron}}} \right)}} \left] { \, + \, } \right[{\text{MTAC}}_{{\left( {{\text{CLX }} + {\text{ Dendron}}} \right)}} /{\text{ MTAC}}_{{({\text{CLX}})}} ]$$

The result was interpreted as synergistic when FICI was $\le$ 0.5, additive when 0.5 $<$ FICI $\le$ 1, indifferent when 1 $<$ FICI $<$ 4 and antagonistic when FICI $\ge$ 4^15^.

### Combined treatment against cysts

Experiments were prepared as explained above for the cysticidal activity assays. Briefly, 100 μL of adjusted cyst inoculum was added to each microtiter plate well. The combination of the most effective dendron in concentrations ranging from 2 to 64 mg/L (MCC) and CLX, in concentrations from 1 to 8 mg/L, were added to each well (50 μL of each compound). After 24 and 48 h of treatment, the compounds were discarded. Wells were then washed twice with 1 × PBS (10x, Sigma Aldrich, St. Louis, MO, USA) and fresh medium was added. Finally, plates were incubated under optimal growing conditions. They were observed three times per week using an inverted microscope (Motic AE21) for 21 days to determine excystment. To determine synergy, the FICI was calculated as previously described.

### Cytotoxicity assay in HeLa cells

HeLa cells were cultured in Dulbecco's Modified Eagle Medium (DMEM) (Gibco) supplemented with 10% fetal bovine serum and 1% of an antibiotic mixture. The inoculum was adjusted to 1 × 10^4^ cells per well in a 24-well microtiter plate. Cells were grown for 3–4 days at 37 °C with 5% CO_2_ to reach confluence^17^. Then, the medium was discarded and 400 μL of the most effective dendron and CLX concentrations diluted in DMEM medium were added.

After 24 and 48 h of treatment, the medium was discarded once again, and the wells were washed twice with 1 × PBS (10x, Sigma Aldrich, St. Louis, MO, USA). After that, 500 μL of fresh medium and 50 μL of 3-(4,5-dimethylthiazol-2-yl)-2,5-diphenyltetrazolium bromide (MTT) was added (Sigma-Aldrich Ldt). The plates were incubated at 37 °C for 4 h. Then, the medium was discarded and 500 μL of DMSO were added^20^. Absorbance values were determined at the 570 nm wavelength using a spectrophotometer (BioTek Instruments Inc. Model: ELX 800).

Viability was calculated as (absorbance mean of treated/absorbance mean of non-treated) × 100. Values higher than 90% viability were considered non-cytotoxic, while values between 75 and 90% were considered low cytotoxicity. If viability descended to the range of 60–75%, it was deemed a moderate cytotoxic level. High cytotoxicity was established when viability values were lower than 60%^21^.

### Scanning electron microscopy (SEM)

SEM study was performed to evaluate the impact of the most effective dendron, CLX and combinations of these as previously described by our group^16^. To perform these studies, 200 μL of adjusted trophozoite suspension were placed on a glass coverslip. After 1 h of incubation under optimal growing conditions (32 °C for *A. polyphaga* 2961 and 37 °C for *A. griffini* MYP2004), the medium was discarded and treated for 24 or 48 h. Then, the medium was discarded, the wells were washed twice with PBS, and the fixative solution (2% glutaraldehyde and 1% CaCl_2_ in Milloning’s solution (NaH_2_PO_4_·H_2_O and NaOH)) was added for 1 h. Afterwards, the coverslips were washed with washing solution. The samples were dehydrated in increasing concentrations of ethanol (30, 50, 70, 95 and 100%) and finally in anhydrous acetone. Desiccation was performed in a critical point drying apparatus (Polaron E-3000), and the coverslips were mounted on aluminium stubs and coated with gold (Polaron E-5000/5100). The samples were examined using a Scanning Electron Microscope (JSM-IT500, JEOL) at the Medicine and Biology Research Support Center (University of Alcalá).

### Statistical analysis

For statistical analysis and graph generation, Microsoft Excel (Microsoft Office 365, Microsoft, Redmond, Washington, USA) and GraphPad Prism 8® (GraphPad Software, San Diego, California, USA) were used. Two-way ANOVA and One-way ANOVA (followed by Dunnett’s multiple comparisons test) were performed, and significance was established at *p* < 0.05. IC_50_ was obtained on GraphPad Prism 8^®^ a using non-linear regression analysis^22^.

## Results

### Amoebicidal activity assays against trophozoites

**Figure. 2:**
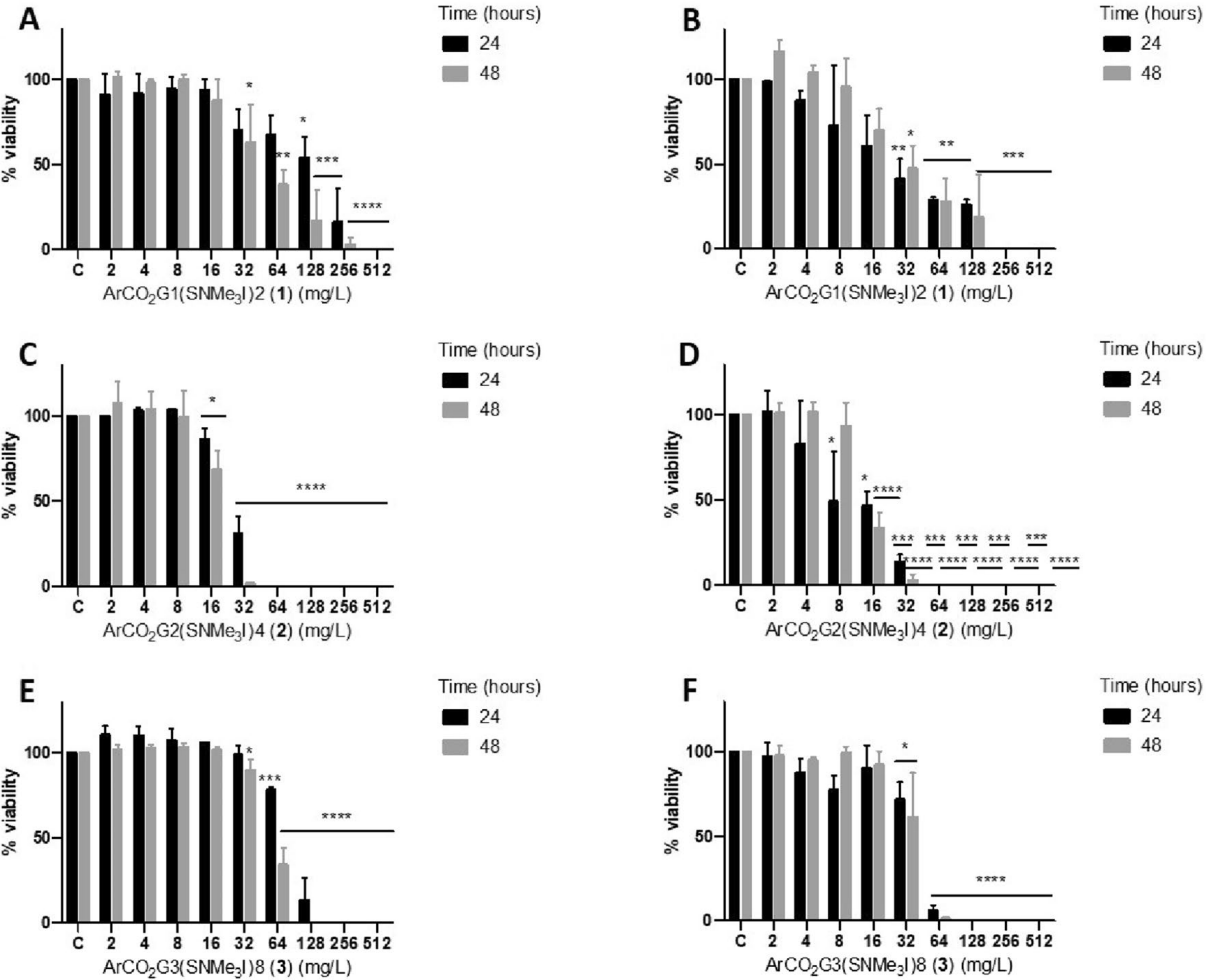
Viability percentage of trophozoites treated with dendritic compounds. *A. griffini* MYP2004: (**A**) ArCO_2_G_1_(SNMe_3_I)_2_ (**1**), (**C**) ArCO_2_G_2_(SNMe_3_I)_4_ (**2**), (**E**) ArCO_2_G_3_(SNMe_3_I)_8_ (**3**). *A. polyphaga* 2961: (**B**) ArCO_2_G_1_(SNMe_3_I)_2_ (**1**), (**D**) ArCO_2_G_2_(SNMe_3_I)_4_ (**2**), (**F**) ArCO_2_G_3_(SNMe_3_I)_8_ (**3**). * *p* $\le$ 0.05; ** *p* $\le$ 0.01; *** *p* $\le$ 0.001; **** *p* $\le$ 0.0001.

All dendrons evaluated, ArCO_2_G_1_(SNMe_3_I)_2_ (**1**), ArCO_2_G_2_(SNMe_3_I)_4_ (**2**) and ArCO_2_G_3_(SNMe_3_I)_8_ (**3**) showed amoebicidal activity against trophozoites, although in variable concentrations (Fig. 2, Table 1). ArCO_2_G_2_(SNMe_3_I)_4_ (2) was the most effective, exhibiting a MTAC of 64 mg/L for *A. griffini* MYP2004 after 24 and 48 h of treatment (Fig. 2, Table 1). In the case of *A. polyphaga* 2961, this concentration was equal after 24 h of treatment and varied in the range of 32–64 mg/L after 48 h of treatment (Fig. 2, Table 1). The IC_50_ of each dendron is shown in Table 2.

**Table 1:** MTAC (mg/L) values in *A. griffini* MYP2004 and *A. polyphaga* 2961 after 24 and 48 h of dendron treatment.

	MTAC (mg/L)			
	*A. griffini* MYP2004		*A. polyphaga* 2961	
Dendrons	24 h	48 h	24 h	48 h
ArCO_2_G_1_(SNMe_3_I)_2_ (**1**)	512	256–512	256	128–256
ArCO_2_G_2_(SNMe_3_I)_4_ (**2**)	64	64	64	32–64
ArCO_2_G_3_(SNMe_3_I)_8_ (**3**)	128–256	128	128	64–128

ArCO_2_G_1_(SNMe_3_I)_2_ (**1**), ArCO_2_G_2_(SNMe_3_I)_4_ (**2**) and ArCO_2_G_3_(SNMe_3_I)_8_ (**3**).

**Table 2:** IC_50_ (mg/L) values in *A. griffini* MYP2004 and *A. polyphaga* 2961 after 24 and 48 h of dendron treatment.

	IC_50_ (mg/L)			
	*A. griffini* MYP2004		*A. polyphaga* 2961	
Dendrons	24 h	48 h	24 h	48 h
ArCO_2_G_1_(SNMe_3_I)_2_ (**1**)	224.50	57.75	23.96	29.41
ArCO_2_G_2_(SNMe_3_I)_4_ (**2**)	26.97	16.66	8.24	11.68
ArCO_2_G_3_(SNMe_3_I)_8_ (**3**)	135.30	72.77	44.60	39.33

ArCO_2_G_1_(SNMe_3_I)_2_ (**1**), ArCO_2_G_2_(SNMe_3_I)_4_ (**2**) and ArCO_2_G_3_(SNMe_3_I)_8_ (**3**).

### Cysticidal activity assays

The MMC of the dendritic compounds on the excystment of cysts was determined by observation under the microscope^18^. Only ArCO_2_G_2_(SNMe_3_I)_4_ (**2**) demonstrated a cysticidal activity in the concentration range tested (2–512 mg/L) (Table 3). For *A. griffini* MYP2004, the MCC was 64 mg/L, while for *A. polyphaga* 2961 it was higher, 128 mg/L (Table 3). There was no change between both times of incubation.

**Table 3:** MCC (mg/L) in *A. griffini* MYP2004 and *A. polyphaga* 2961 after 24 and 48 h of treatment of dendron treatment

	MCC (mg/L)			
	*A. griffini* MYP2004		*A. polyphaga* 2961	
Dendrons	24 h	48 h	24 h	48 h
ArCO_2_G1(SNMe_3_I)2 (**1**)	> 512	> 512	> 512	> 512
ArCO_2_G2(SNMe_3_I)4 (**2**)	64	64	128	128
ArCO_2_G3(SNMe_3_I)8 (**3**)	> 512	> 512	> 512	> 512

ArCO_2_G_1_(SNMe_3_I)_2_ (**1**), ArCO_2_G_2_(SNMe_3_I)_4_ (**2**) and ArCO_2_G_3_(SNMe_3_I)_8_ (**3**).

### Combined treatment against trophozoites

**Figure 3:**
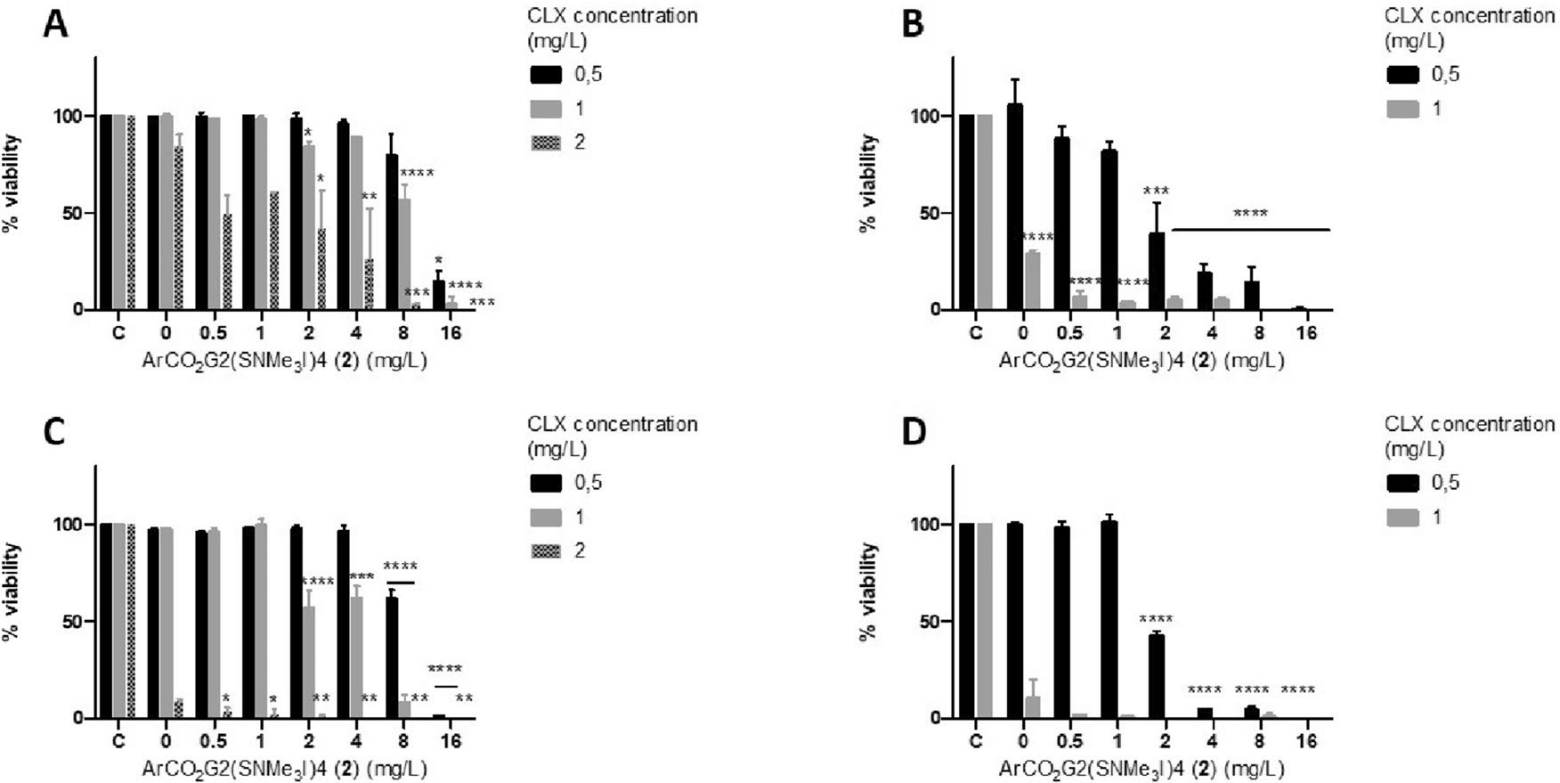
Viability percentage of trophozoites after combination treatments with (ArCO_2_G_2_(SNMe_3_I)_4_ (**2**) and CLX. (**A**) *A. griffini* MYP2004 24 h treatment, (**B**) *A. polyphaga* 2961 24 h treatment, (**C**) *A. griffini* MYP2004 48 h treatment, (**D**) *A. polyphaga* 2961 48 h treatment. * *p* ≤ 0.05; ** *p* ≤ 0.01; *** *p* ≤ 0.001; **** *p* ≤ 0.0001.

The effective concentrations used in the combination treatments were lower than the effective concentrations for each compound tested individually. Moreover, when the FICI was calculated, a synergistic effect was found when 16 mg/L ArCO_2_G_2_(SNMe_3_I)_4_ (**2**)/1 mg/L CLX were combined against *A. griffini* trophozoites, and 16 mg/L ArCO_2_G_2_(SNMe_3_I)_4_ (**2**)/0.5 mg/L CLX against *A.* *polyphaga* trophozoites. Therefore, the ArCO_2_G_2_(SNMe_3_I)_4_ (**2**) effective concentration was reduced from 64 to 16 mg/L and the CLX effective concentration was reduced from 2–4 mg/L to 0.5–1 mg/L (non-cytotoxic concentrations). Under these conditions, the combined therapy improved compound efficacy and reduced the effective concentrations and thus, the cytotoxicity of both compounds. Nevertheless, some of the other effective combinations had additive or indifferent effects (Tables 4 and 5, Fig. 3).

**Table 4:** FICI in *A. griffini* MYP2004 trophozoites and interpretation of the effect

	*A. griffini* MYP 2004					
	MTAC (mg/L)				FICI	Interpretation
	Alone		In combination			
Time (hours)	ArCO_2_G_2_(SNMe_3_I)_4_ (2)	CLX	ArCO_2_G_2_(SNMe_3_I)_4_ (2)	CLX		
24	64	4	16	2	0.75	Additive
	64	4	8	4	1.13	Indifferent
48	64	4	16	1	0.50	Synergy
	64	4	4	2	0.56	Additive

MTAC (mg/L) for ArCO_2_G_2_(SNMe_3_I)_4_ (**2**) and CLX alone or in combination after 24 and 48 h of treatment.

**Table 5:** Fractional inhibitory concentration index (FICI) in *A. polyphaga* 2961 trophozoites and interpretation of the effect

	*A. polyphaga* 2961					
	MTAC (mg/L)				FICI	Interpretation
	Alone		In combination			
Time (hours)	ArCO_2_G_2_(SNMe_3_I)_4_ (2)	CLX	ArCO_2_G_2_(SNMe_3_I)_4_ (2)	CLX		
24	64	2	16	0.5	0.50	Synergy
	64	2	8	1	0.63	Additive
48	32–64	2	4	1	0.56–0.63	Additive
	32–64	2	2	1	0.53–0.56	Additive

MTAC (mg/L) for ArCO_2_G_2_(SNMe_3_I)_4_ (**2**) and CLX alone or in combination after 24 and 48 h of treatment.

### Combined treatment against cysts

As described in the combination assay against trophozoites, effective treatment concentrations against cysts were lower in combination than for each of the drugs individually. FICI values showed a synergistic effect for the highest ArCO_2_G_2_(SNMe_3_I)_4_ (**2**) concentrations tested in combination. For example, synergy was found when 16 mg/L ArCO_2_G_2_(SNMe_3_I)_4_ (**2**)/2 mg/L CLX were combined against *A. griffini* cysts, and 32 mg/L ArCO_2_G_2_(SNMe_3_I)_4_ (**2**)/2 mg/L CLX against *A. polyphaga* cysts. Therefore, the ArCO_2_G_2_(SNMe_3_I)_4_ (**2**) effective concentration was reduced from 64–128 to 16–32 mg/L and the CLX effective concentration was reduced from 8 to 1–2 mg/L. Under these conditions, the combined therapy improved compound efficacy and reduced the effective concentrations against cysts and, in consequence, the cytotoxicity of both compounds. However, the primary outcome was an additive effect, once again (Tables 6 and 7).

**Table 6:** FICI in cysts and its interpretation in *A. griffini* MYP2004. MCC (mg/L) for ArCO_2_G_2_(SNMe_3_I)_4_ (**2**) and CLX alone or in combination after 24 and 48 h of treatment.

	*A. griffini* MYP 2004					
	MCC (mg/L)				FICI	Interpretation
	Alone		In combination			
Time (hours)	ArCO_2_G_2_(SNMe_3_I)_4_ (2)	CLX	ArCO_2_G_2_(SNMe_3_I)_4_ (2)	CLX		
24	64	8	32	2	0.75	Additive
	64	8	16	4	0.75	Additive
48	64	8	32	1	0.63	Additive
	64	8	16	2	0.50	Additive

**Table 7:** FICI in cysts and its interpretation in *A. polyphaga* 2961

	*A. polyphaga* 2961					
	MCC (mg/L)				FICI	Interpretation
	ALONE		IN COMBINATION			
Time (hours)	ArCO_2_G_2_(SNMe_3_I)_4_ (2)	CLX	ArCO_2_G_2_(SNMe_3_I)_4_ (2)	CLX		
24	128	8	64	1	0.63	Additive
	128	8	32	2	0.50	Sinergy
48	128	8	32	1	0.38	Sinergy
	128	8	4	4	0.53	Additive

MCC (mg/L) for ArCO_2_G_2_(SNMe_3_I)_4_ (**2**) and CLX alone or in combination after 24 and 48 h of treatment.

### Cytotoxicity assay in HeLa cells

The cytotoxicity of treatment combinations with amoebicidal and amoebostatic activity was evaluated in HeLa cells. Individual evaluation of ArCO_2_G_2_(SNMe_3_I)_4_ (**2**) and CLX cytotoxicity in HeLa cells performed previously showed that ArCO_2_G_2_(SNMe_3_I)_4_ (**2**) demonstrated low cytotoxicity in the range of 4–8 mg/L, according to the aforementioned criteria, even after 48 h of exposure^23^. However, CLX exhibited high cytotoxicity at similar concentrations after only 24 h.

**Figure 4:**
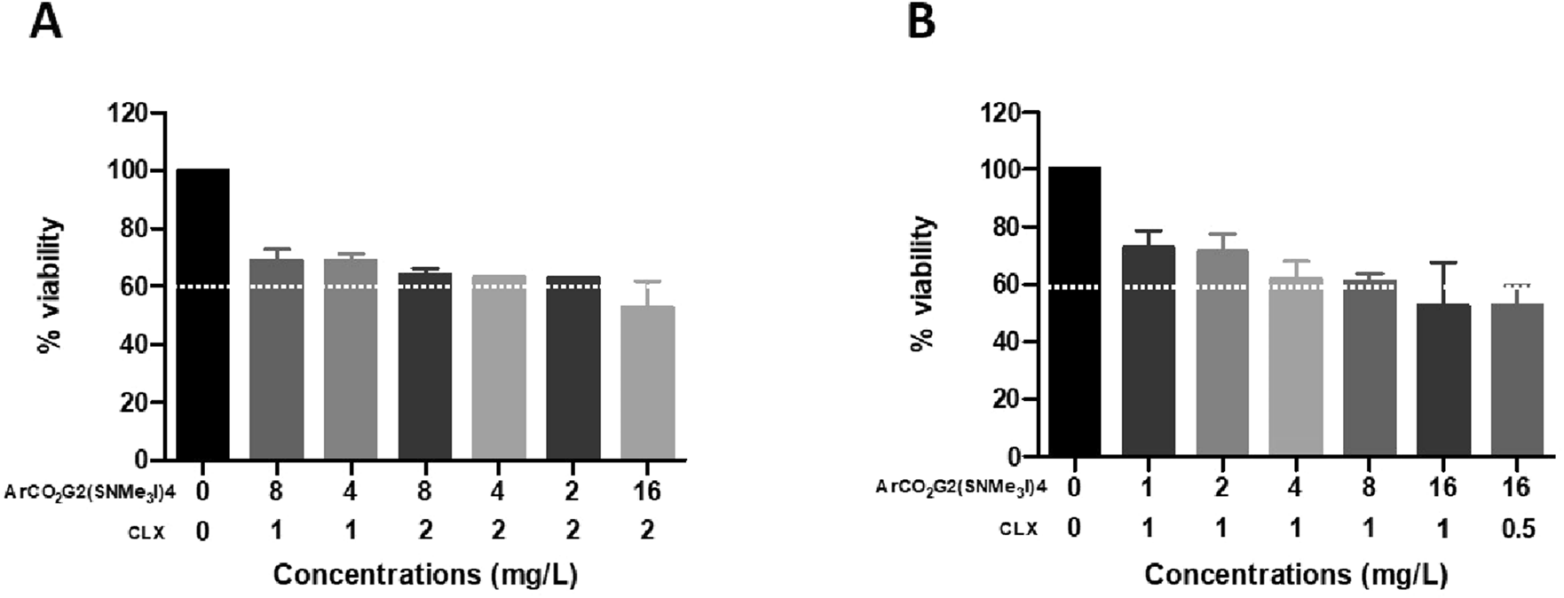
Cytotoxicity of treatment combinations. Percentage of viable HeLa cells after treatment with each of the selected combinations of ArCO_2_G_2_(SNMe_3_I)_4_ (**2**) and CLX effective against trophozoites and cysts. (**A**) Cellular viability after 24 h of treatment. (**B**) Cellular viability after 48 h of treatment.

Effective amoebicidal combinations against trophozoites of both strains showed some cytotoxicity. Nevertheless, the combination that showed a moderate cytotoxicity (65% cell viability) could inhibit proliferation and eliminate 100% *A. polyphaga* 2961 while reducing *A. griffini* MYP2004 viability up to 97% after a 24 h treatment (8 mg/L ArCO_2_G_2_(SNMe_3_I)_4_ (**2**)/2 mg/L CLX) (Fig. 4, Table 8). In addition, the combination of 2 mg/L ArCO_2_G_2_(SNMe_3_I)_4_ (**2**) and 1 mg/L CLX eliminated 100% of *A. polyphaga* trophozoites after a 48 h treatment and showed moderate toxicity (75% cell viability) (Fig. 4, Table 8).

**Table 8:** Viability (%) in trophozoites of *A. griffini* MYP2004 and *A. polyphaga* 2961 after 24 and 48 h of treatment with each of the effective concentrations evaluated in the cytotoxicity assay.

		Combinations of ArCO_2_G_2_(SNMe_3_I)_4_ (2) (mg/L) + CLX (mg/L)					
		16 + 2	8 + 2	8 + 1	4 + 2	4 + 1	2 + 2
% Viability (24 h)	*A. griffini* MYP2004	0%	3%	56%	26%	89%	42%
	*A. polyphaga* 2961	0%	0%	0%	0%	5%	0%
		16 + 1	16 + 0.5	8 + 1	4 + 1	2 + 1	1 + 1
% Viability (48 h)	*A. griffini* MYP2004	0%	1%	9%	62%	57%	100%
	*A. polyphaga* 2961	0%	0%	0%	0%	0%	1%

The effective treatment concentrations achieved in different combinations against cysts were higher than the ones used against trophozoites that showed high cytotoxicity. Therefore, further cytotoxicity was not evaluated, as it was assumed to be greater than in those treatment combinations previously studied, i.e., more cytotoxic than the combinations of concentrations that were effective against trophozoites.

### Scanning electron microscopy (SEM)

**Figure 5:**
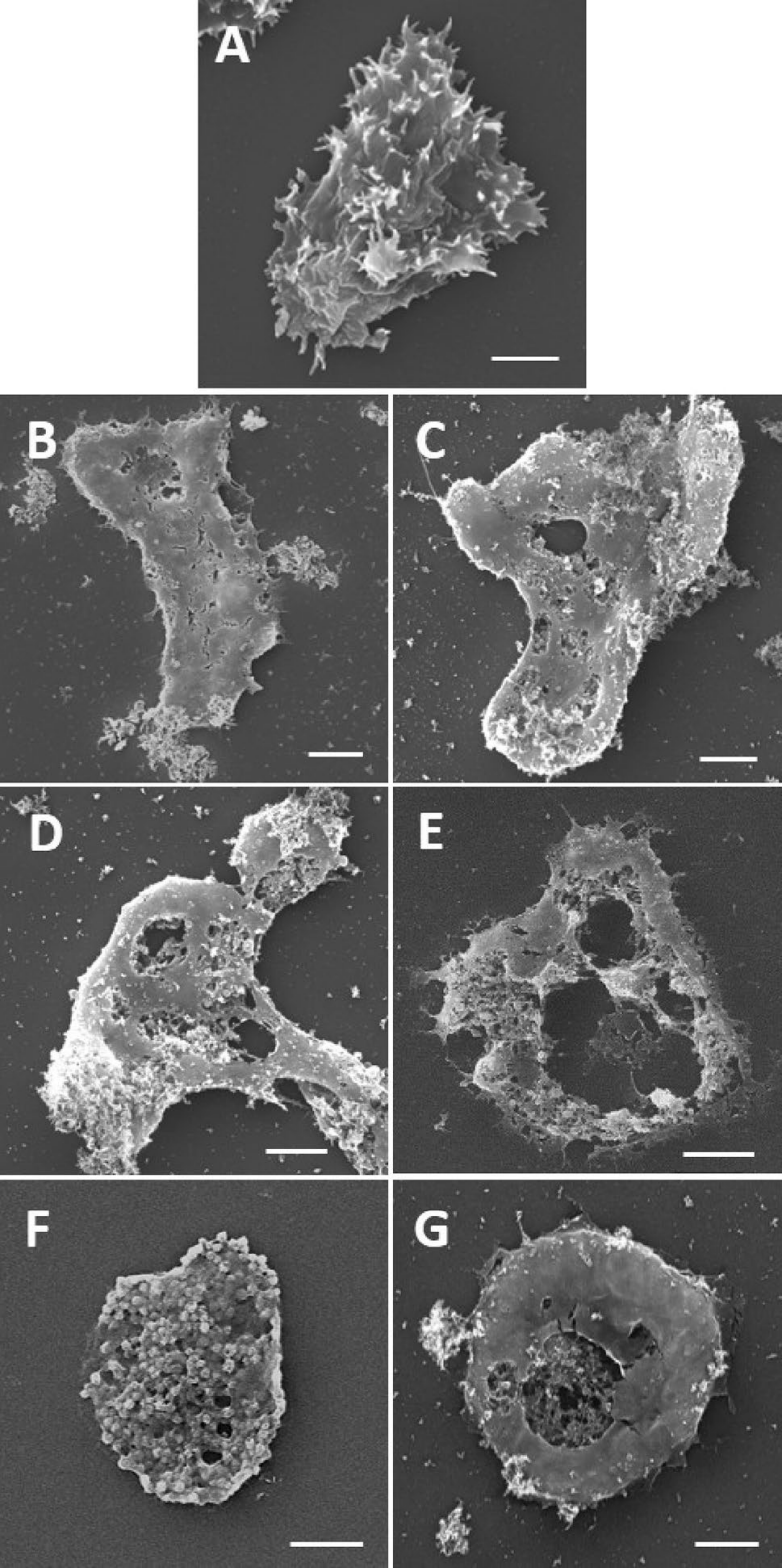
Images of *A. griffini* MYP2004 obtained by SEM after 24 or 48 h of treatment. (**A**) Untreated control, (**B**) 32 mg/L of ArCO_2_G2(SNMe_3_I)_4_ (**2**) after 24 h, (**C**) 16 mg/L of ArCO_2_G2(SNMe_3_I)_4_ (**2**) after 48 h, (**D**) 2 mg/L of CLX after 24 h, (**E**) 2 mg/L of CLX after 48 h, (**F**) 8 mg/L of ArCO_2_G2(SNMe_3_I)_4_ (**2**) and 1 mg/L of CLX after 24 h, (**G**) 8 mg/L of ArCO_2_G2(SNMe_3_I)_4_ (**2**) and 0.5 mg/L of CLX after 48 h. Scale bars = 5 µm.

**Figure 6:**
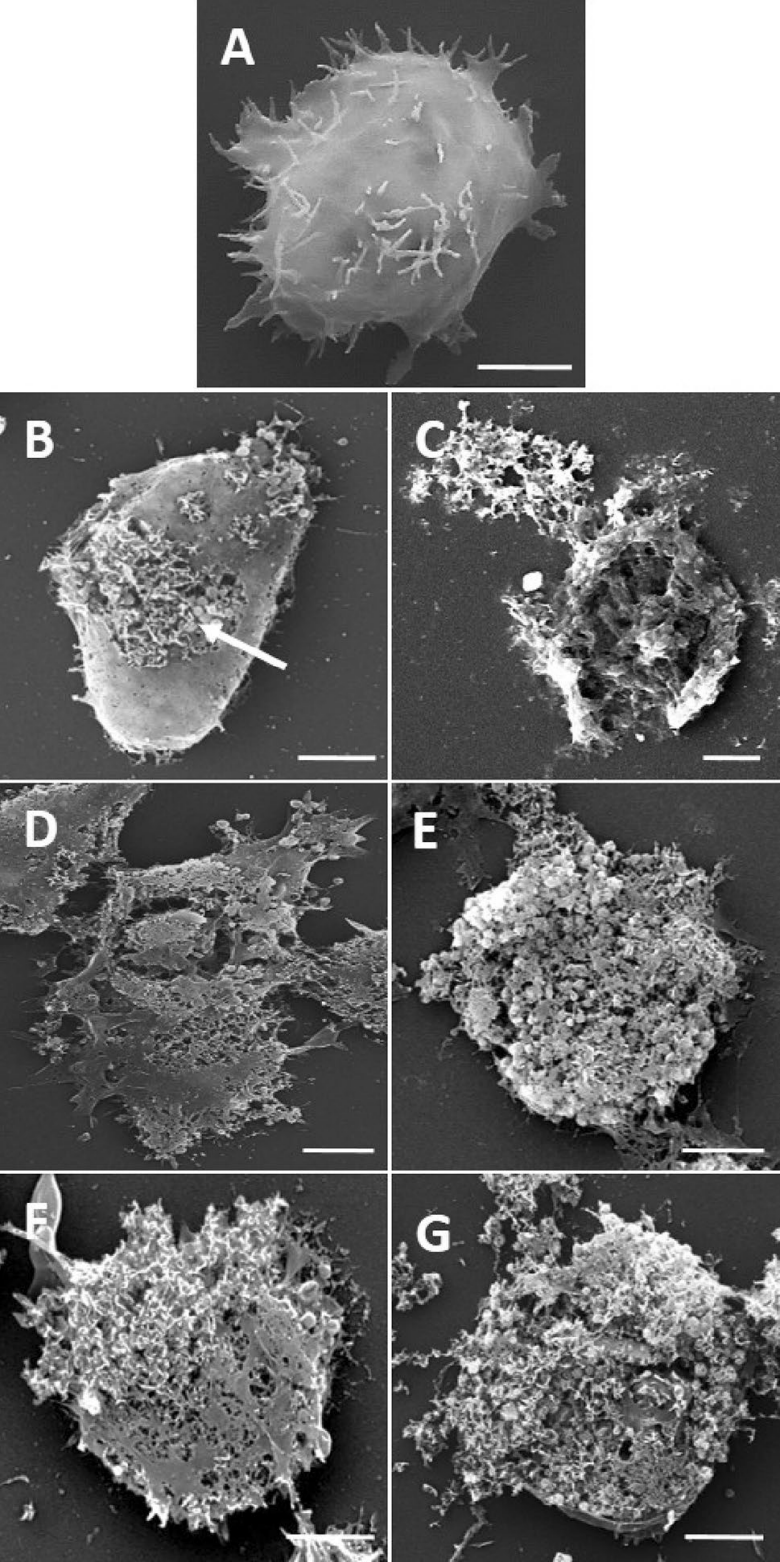
Images of *A. polyphaga* 2961 obtained by SEM after 24 or 48 h of treatment. (**A**) Untreated control, (**B**) 16 mg/L of ArCO_2_G2(SNMe_3_I)_4_ (**2**) after 24 h, **C)** 16 mg/L of ArCO_2_G2(SNMe_3_I)_4_ (**2**) after 48 h, (**D**) 1 mg/L of CLX after 24 h, (**E**) 1 mg/L of CLX after 48 h, **F)** 2 mg/L of ArCO_2_G2(SNMe_3_I)_4_ (**2**) and 0.5 mg/L of CLX after 24 h, (**G**) 2 mg/L of ArCO_2_G2(SNMe_3_I)_4_ (**2**) and 0.5 mg/L of CLX after 48 h. Scale bars = 5 µm.

Images of untreated trophozoites obtained by SEM showed the characteristics of healthy amoebae with numerous acanthopodia and membrane integrity. However, membrane alterations were observed when the amoebae were treated with both the ArCO_2_G2(SNMe_3_I)_4_ (**2**) dendron and CLX, as monotherapy and in combination (Figs. 5 and 6).

As observed in the images, CLX completely disintegrates the *Acanthamoeba* membrane, causing the release of cytoplasmic constituents (Figs. 5D,E, 6D,E), reducing the number of acanthopodia and inducing a rounded shape in trophozoites (Figs. 5D,E, 6D,E). These observations may suggest that dendron ArCO_2_G_2_(SNMe_3_I)_4_ (**2**) caused the appearance of holes, possibly due the interaction with the negative charges on the amoeba surface and, in consequence, it may produces the disruption of the plasma membrane (Fig. 6B (arrow)). In addition, after treatments with drug combinations, no other changes were noticed apart from the ones aforementioned for each of the treatments as monotherapies (Figs. 5F,G, 6F,G). Alterations were more evident after 48 h of treatment.

## Discussion

AK is a rare, sight-threatening disease, although the severity of its outcomes together with the lack of effective treatments has made the development of new therapeutic agents an urgent line of research^3,24^.

On the one hand, CLX is a biguanide considered a standard treatment drug in AK cases. It can be used in combination with a diamidine or as a monotherapy^24,25^. However, it is highly cytotoxic at effective concentrations^26,27^. To solve this problem, combination strategies have been proposed^15^. On the other hand, dendritic compounds have shown effectiveness in previous studies not only against bacteria and viruses, but also against amoebae^17,28–30^. Moreover, these compounds are water-soluble, and their structure facilitates a directed design, characteristics that make them suitable therapeutic agents^10^. Their cationic structure, like CLX, may explain their biocidal activity, even though their mode of action has not yet been completely elucidated^31^. The three dendrons evaluated, ArCO_2_G_1_(SNMe_3_I)_2_ (**1**), ArCO_2_G_2_(SNMe_3_I)_4_ (**2**) and ArCO_2_G_3_(SNMe_3_I)_8_ (**3**), were effective against trophozoites at different concentrations. In addition, the second generation dendron, ArCO_2_G_2_(SNMe_3_I)_4_ (**2**), with four positive charges on the periferial groups and PBA at the focal point showed cysticidal activity. Cysts have the ability to resist treatment by different compounds because they form a protective wall, composed mainly of cellulose (endocyst), proteins and polysaccharides (exocyst)^32^. Nevertheless, *A. griffini* MYP2004 cysts seemed to be more sensitive than *A. polyphaga* 2961 cysts to treatment with ArCO_2_G_2_(SNMe_3_I)_4_ (**2**). Although this result may seem to be contradicted by the data shown in literature, the dendron could act differentially on each cyst wall depending on its exact composition^3^. Previous studies described differences in cystic proteins within isolates, so these data may also indicate protein variability between the *Acanthamoeba* strains used in our study and other amoebic strains^33^.

CLX, which caused the alterations observed by SEM after treatments, is a cationic molecule that interacts with the negatively charged plasma membrane, thus provoking membrane destabilization, pore formation, cytoplasmic content leakage and cell death^14,34^. Dendrons are also cationic molecules, thus their mode of action might be similar and alterations may be due to positive charges that interact with and disrupt cell structures^31^. In addition, dendrons have an aromatic ring at their focal point (growing dendron point where peripherial groups emerge) that could improve their antibacterial activity^35^.

In previous studies, amoebicidal activity against *A. polyphaga* 2961 trophozoites and cysts shown by dendritic wedge topology systems with isobutyric acid at the focal point (IC_50_ at 24 h treatment: ArCO_2_G2(SNMe_3_I)4 for trophozoites 8.24 mg/L and for cysts 128 mg/L) was greater than that shown by spherical topology systems containing a polyphenoxy nucleus (IC_50_ at 24 h treatment: G1O_3_(SNMe_3_I)_6_ for trophozoites 16.9 mg/L and for cysts > 512 mg/L) or a silicon atom nucleus (IC_50_ 24 h G1O_3_(SNMe_3_I)_6,_ 430.1 mg/L for trophozoites and > 512 mg/L for cysts)^36^*.* In view of these results and considering that they all have a similar number of positive charges in their structure, we suggest that the presence of the aromatic ring modulates the activity of the dendritic system, and the topology of the system also seems to have a clear influence on the activity. The dendritic wedge presents a more open structure that could provide the aromatic ring with the possibility for interacting with the amoeba membrane*.*

The FICI index was calculated in the combination assays^15,37^. The main goal was to find a combination that could reduce effective doses to less cytotoxic ones. To this end, the most effective dendron ArCO_2_G_2_(SNMe_3_I)_4_ (**2**) was tested in combination with CLX. The results showed a synergistic effect at highly cytotoxic concentrations, although other combinations with additive effects achieved a reduction in the effective concentration and had moderate cytotoxicity. For example, certain combinations were able to reduce *A. griffini* MYP2004 viability to only 3% and completely eliminate *A. polyphaga* 2961 trophozoites (0% viability). Consequently, we highlight those combinations which achieved a reduction in effective concentrations of CLX with an increase in amoebicidal activity and a significant reduction in cell cytotoxicity. We also propose that the use of these combinations in prevention and treatment strategies could be studied in in vivo models. Additionally, combined therapy has been shown to prevent the development of new resistant strains, thus combination strategies may have extra value in the fight against AK resistance^15^. In summary, our data support the suitability of these promising dendrons alone and in combination for treating AK.

## Conclusions

The cationic dendrons with isobutyric acid at the focal point evaluated in this study showed amoebicidal activity against trophozoites. Furthermore, the second generation dendron (ArCO_2_G2(SNMe_3_I)_4_) (**2**) with four positive charges on the surface had cysticidal activity and a synergistic effect when combined with CLX, a standard treatment drug. Besides, the combinations with a drastic reduction in trophozoite viability, even to 0%, showed only moderate cytotoxicity. Additionally, the ArCO_2_G2(SNMe_3_I)_4_ (**2**) dendron and CLX modes of action showed similarities as both were capable of disrupting trophozoite membranes.

## Data Availability

The datasets used and/or analysed during the current study available from the corresponding author on reasonable request.
